# Antibody Format and Serum Disposition Govern Ocular Pharmacokinetics of Intravenously Administered Protein Therapeutics

**DOI:** 10.3389/fphar.2021.601569

**Published:** 2021-05-06

**Authors:** Vittal Shivva, C. Andrew Boswell, Hanine Rafidi, Robert F. Kelley, Amrita V. Kamath, Susan R. Crowell

**Affiliations:** ^1^ Preclinical and Translational Pharmacokinetics and Pharmacodynamics, Genentech, South San Francisco, CA, United States; ^2^ Pharmaceutical Development, Genentech, South San Francisco, CA, United States

**Keywords:** ocular pharmacokinetics, intravenous administration, rabFab, rabIgG, aqueous humor, vitreous humor, tissue partitioning

## Abstract

Protein therapeutics have witnessed tremendous use and application in recent years in treatment of various diseases. Predicting efficacy and safety during drug discovery and translational development is a key factor for successful clinical development of these therapies. In general, drug related toxicities are predominantly driven by pharmacokinetic (PK) exposure at off-target sites. This work explores the ocular PK of intravenously administered protein therapeutics to understand impact of antibody format on off-site exposure. Species matched non-binding rabbit antibody proteins (rabFab and rabIgG) were intravenously administered to male New Zealand White rabbits at a single 1 mg bolus dose and exposure was measured up to 3 weeks. As anticipated based on absence of FcRn recycling, rabFab has relatively fast systemic PK (CL–943 mL/day and t_1/2_–1.93 days) compared to rabIgG (CL–18.5 mL/day and t_1/2_–8.93 days). Similarly, rabFab has lower absolute ocular exposure in ocular compartments (e.g., vitreous and aqueous humor) compared to rabIgG, despite higher relative exposures (measured as percent tissue partition in ocular tissues relative to serum, based on C_max_ and AUC). In general, percent tissue partition based on AUC (in aqueous and vitreous humor) relative to serum exposure were 10.4 and 8.62 for rabFab respectively and 1.11 and 0.64 for rabIgG respectively. This work emphasizes size and format based ocular exposure of intravenously administered protein therapeutics. Findings from this work enable prediction of format based ocular exposure for systemically administered antibody based therapeutics and aid in selection of molecule format for clinical candidate to minimize ocular exposure.

## Introduction

Monoclonal antibodies (mAbs) and antibody-based therapeutics have emerged as mainstay of drug approvals in recent years (Kaplon et al., 2020; Lu et al., 2020). Similar to other drug modalities, development of therapeutic antibodies comes with challenges such as establishing efficacy, safety and monitoring and managing toxicities. Several targeted antibodies for oncology indications with target expression in the eye and many antibody drug conjugates (ADCs) with cytotoxic payload have reported to cause ocular toxicity (Renouf et al., 2012; Eaton et al., 2015). In general, intravenously (IV) administered biologic drugs have been assumed to exhibit little to no partitioning into the eye due to various barriers and impediments. Typically, eye is considered an immune privileged site with a variety of static barriers, including layers of cornea, sclera, and retina, blood-aqueous and blood–retinal barriers as well as dynamic barriers such as choroidal, conjunctival blood flow, lymphatic clearance and tear dilution that prevent immune responses and preserve vision under normal homeostatic conditions (Perez et al., 2013). The regional immune system in the eye preserves immunosuppressive microenvironment and maintains vision by regulating innate and adaptive immune status of the eye (Keino et al., 2018). Ocular immune privilege is mainly mediated by regulatory T cells, which are generated by the anterior chamber-associated immune deviation, and ocular resident cells including corneal endothelial cells, retinal pigment epithelial cells, and aqueous humor that encounter cytotoxic effector T cells under normal homeostatic conditions (Mochizuki et al., 2013; Keino et al., 2018).

Despite the eye being an immune privileged site, many systemically administered targeted therapies have shown to elicit ocular toxicities. Additionally, many ADCs were shown to induce ocular toxicities following IV administration in non-clinical species and in the clinic; the nature of these toxicities have been generally attributed to non-specific biodistribution of ADCs into ocular tissues and payload mediated toxicities (Eaton et al., 2015; Donaghy, 2016). Commonly observed ocular toxicities following IV administration of antibody based targeted anti-cancer therapies and ADCs include iris irritation, dry eyes, conjunctivitis, loss of visual acuity, hyper-lacerations, episcleritis, glaucoma and uveitis to name a few (Renouf et al., 2012; Ho et al., 2013; Eaton et al., 2015; Donaghy, 2016; Fu et al., 2017). Target protein/antigen expression in the eye drives pathogenesis in many of these cases. Ocular partitioning of systemically administered antibodies and/or downstream target engagement in the eye can lead to disruption of ocular barriers, create imbalance in the delicate homeostatic microenvironment and may lead to adverse events (Renouf et al., 2012; Ho et al., 2013). Such examples include cetuximab, ipilimumab, panitimumab, pertuzumab, and rituximab where toxicities were mostly reported to be minor and common adverse events while some patients on ipilimumab (<1%) have reported to experience severe adverse events such as episcleritis, blindness (Fu et al., 2017).

Intravitreal (ITV) drug administration is the most common route for local ocular drug delivery for retinal diseases (Morlet et al., 1993). Several biologics targeting vascular endothelial growth factor (VEGF) for the treatment of retinal diseases are administered as ITV injections (Jin and Hwang, 2017; Yannuzzi and Freund, 2019). Though ocular and systemic pharmacokinetics (PK) of mAbs following ITV administration have been well documented in preclinical models and in human (Gadkar et al., 2015; Avery et al., 2017; Crowell et al., 2019; Caruso et al., 2020), ocular PK post IV dosing of mAbs is not well characterized. Limited efforts in this space include empirical compartmental modeling to predict ocular PK of small molecules post IV administration in rabbits as described in Vellonen et al. (Vellonen et al., 2016) and ocular PK of bevacizumab in one eye after ITV dosing in other eye of rabbit, using systemic PK as driver for ocular exposure as described in Bakri et al. (Bakri et al., 2007b).

To the best of our knowledge, ocular PK of mAbs and antibody-based therapies following IV administration have not been explored adequately. We believe addressing this knowledge gap in the literature can help understand ocular exposure and subsequently in predicting exposure based ocular activity of systemically administered protein therapeutics. Additionally, knowledge on ocular exposure related to design characteristics would aid in molecule design and format selection of novel biologics, to minimize ocular exposure. To investigate ocular PK post IV injection, we used two most commonly used formats of protein therapeutics i.e., intact IgG and antibody Fab fragment in rabbit as the *in vivo* model. This IgG and Fab (of the same IgG) were derived from a rabbit antibody campaign and are specific against a 14-mer phosphorylated peptide derived from the intracellular domain of the human cMet receptor (Shatz et al., 2016). Since the antibody is specific for the phospho-Tyr form of the peptide it only recognizes the ligand-activated receptor. This IgG and Fab are not expected to undergo target mediated drug disposition (TMDD) since they are targeted against intracellular protein. In addition, there is a two amino acid insertion in this peptide derived from rabbit cMet such that the antibody should not bind to the rabbit intracellular domain. Given that the IgG and Fab are comprised of rabbit antibody domains they are expected to have minimal potential for inducing immunogenicity in rabbit, allowing us for better investigation of serum and ocular PK. The overall objectives of this research were 1) to characterize ocular and systemic PK of two antibody based drug formats (rabFab and rabIgG) following a single IV bolus dose to New Zealand White rabbits, 2) understand the impact of molecular size and format on systemic and ocular exposure post IV dosing and 3) complement our current understanding of PK of mAbs following IV and ITV administration and enable development of antibody-based systemic therapies with lower potential for ocular exposure.

## Methods

### Animals and Test Articles

This study was conducted at MPI Research (Mattawan, MI, USA) 1) in accordance with Standard Operating Procedures (SOPs) and the protocol as approved by Genentech Inc., (South San Francisco, CA, United States) and 2) in compliance with the requirements contained in the MPI Research Radioactive Materials License. All animals were treated and handled in accordance with the Animal Welfare Act, the Guide for the Care and Use of Laboratory Animals, and the ARVO Statement for the Use of Animals in Ophthalmic and Vision Research. Rabbit was chosen as the test system since it is the most commonly used preclinical species for evaluating ocular pharmacokinetics of biologics (Del Amo and Urtti, 2015; Ahn et al., 2016; Del Amo et al., 2017). Species-matched non-binding rabbit antibody fragment (rabFab; molecular weight 48 kDa, hydrodynamic radius 2.5 ± 0.2 nm) and rabbit immunoglobulin G (rabIgG; molecular weight 150 kDa, hydrodynamic radius 4.86 ± 0.16 nm) produced at Genentech Inc., were utilized as test articles for the PK study (Shatz et al., 2016). This antibody (G10; anti-phospho cMet) is directed against an intracellular antigen that is not available to mediate antigen-dependent clearance (Shatz et al., 2016). Test compounds were radiolabeled with Iodine-125 (^125^I) via the indirect iodination addition method as previously reported (Chizzonite et al., 1991). The ^125^I-radiolabeled protein was purified using NAP5™ desalting columns pre-equilibrated in PBS. The radiolabeled antibodies were shown to be intact by size-exclusion HPLC with no evidence of aggregation or degradation. The specific activities (i.e. ratios of radioactive concentration to protein concentration) for ^125^I-labeled rabFab and rabIgG within dosing solutions were 109 and 107 µCi/mg, respectively; these values were used to convert radioactive concentrations within serum or ocular matrices (e.g. µCi/g or µCi/mL) into mass equivalent protein concentrations (e.g. ng-eq/g or ng-eq/mL).

### Pharmacokinetic Study

Male New Zealand White rabbits (*n* = 24) of approximately 5 months age, weighing 2.6–3.0 kg were randomly assigned to two study groups. Prior to dosing, the animals were sedated by intramuscular administration of acepromazine (1 mg/kg). A single 1 mg dose of either ^125^I rabFab or ^125^I rabIgG was administered as an intravenous bolus injection into the ear vein of rabbits.

Ocular tissue samples for radioactivity analysis were collected at euthanasia at designated time points (at 1 h, 12 h, 1, 2, 4 and 7 days for rabFab and at 1 h, 24 h, 4, 7, 14, and 21 days for rabIgG). The experimental design in this study (number of animals and sample collection time points) was as per the recommendation made by Del Amo and Urtti for assessing ocular PK in rabbits post intravitreal dosing (Del Amo and Urtti, 2015). Though we tested 2 animals per time point, the design allowed 4 replicate eyes (2 eyes per animal) per time point that enabled us testing process-specific variability between samples such as sample collection, processing errors and analytical variability. Specifically, left and right eyes of each animal were collected (two animals per time point). Aqueous humor, vitreous humor, retina-choroid, optic nerve, and ocular remnants (which include all remaining ocular tissues such as cornea, iris, lens and sclera) were collected from the right and left eye of each animal. The eye was frozen in liquid nitrogen for approximately 15–20 s and placed on dry ice or stored frozen at −60 to −90°C for at least 2 h, but no more than 4 days. The ocular globe was enucleated, and all the aqueous humor was withdrawn using a tuberculin syringe and placed into a 2-mL polypropylene vial on dry ice. Using a hemostat, the optic nerve was clamped and the whole ocular globe was flash-frozen by submerging into liquid nitrogen for 15–20 s. The globe was placed on a bed of crushed dry ice and, using a scalpel blade, an incision was made through the sclera at approximately 2–3 mm from the limbus and the whole anterior chamber thus removed. Frozen vitreous humor was collected using a scalpel blade to make an incision through the sclera; the sclera and additional tissues were removed from the vitreous humor and dissected over a dry ice bath, before ultimately being placed in a 2-mL polypropylene vial on dry ice. This procedure was repeated for each eye harvesting time point. This procedure has been used successfully in the past with no apparent impact on antibody and fragment stability (Bakri et al., 2007a; Bakri et al., 2007b; Gadkar et al., 2015). Additionally, 1 mL of blood was collected from all surviving animals at 10 min, 1, 6, 12, 24 h, 2, 4, 7, 14, and 21 days post-dose, as well as from all animals at euthanasia. Blood samples were processed to obtain serum for determining systemic exposure using radioactivity analysis. In addition to ocular tissues, other extravascular tissues such as lung, brain, liver, kidney, spleen, lymph node (mandibular), stomach, small intestine, large intestine, gastric muscle, inguinal fat pad, skin and heart were also collected from euthanized animals for radioactivity analysis.

All samples were counted for radioactivity on a gamma counter (Wallac Wizard^®^ 1470 Gamma Counter). Specifically, aqueous humor, vitreous humor, retina-choroid and optic nerve samples were thawed and used for counting radioactivity. Ocular remnants and other tissues such as brain, heart, kidney, liver, spleen, and stomach were separated into different pools and homogenized prior to measurement of radioactivity. The radioactivity level in each sample was converted to concentrations (nanogram-equivalents/milliliter; ng-eq/mL) using specific activity (expressed in µCi/mg). Additionally, an aliquot (approximately 10–20 µL) of serum was counted for radioactivity, before and after processing by trichloroacetic acid (TCA) precipitation. In order to account for accurate protein bound ^125^I counts in serum, and to avoid interference of free ^125^I and low molecular weight ^125^I containing catabolites, serum concentration data were corrected for protein-unbound fraction from TCA precipitation data. The TCA-corrected protein-bound serum concentration data were used for systemic PK estimation purposes. However, TCA precipitation data in this study was limited to serum samples only whereas ocular and other tissue samples were not subjected for TCA precipitation.

### Pharmacokinetic and Statistical Analysis

Serum and tissue concentration-time data were used to estimate pharmacokinetic parameters using Phoenix WinNonlin^®^ version 6.4 (Certara United States, Inc., Princeton, NJ). A non-compartmental PK analysis (NCA) approach consistent with the IV bolus (for serum PK) and extravascular route of administration (for ocular tissue PK) were used for PK parameter estimation. Nominal doses and nominal sampling times were used in PK analysis. For ocular tissue PK, each eye was treated as an individual sample, yielding *n* = 4 samples per time point (2 animals per time point). The individual serum and composite ocular tissue concentration-time data were used for PK calculations. Uniform weighting and the Linear-Trapezoidal (Linear/Log Interpolation) rule were used to estimate the area under the concentration−time curve (AUC). Additionally, other PK parameters such as maximum observed concentration (C_max_), time of maximum observed concentration (T_max_), area under the concentration−time curve from Time 0 to the last measurable time point (AUC_0-t_), area under the concentration−time curve from Time 0 to infinity (AUC_0-∞_), clearance (CL or CL/F, where F is bioavailability fraction in ocular tissues), volume of distribution (V_z_ or V_z_/F) and terminal elimination half-life (t_1/2_) were estimated when possible. Standard errors (SE) were reported for the mean PK parameter values where applicable. PK parameters for ocular tissues were reported when sufficient data was available to derive parameters. Means and standard deviations (SD) were calculated for serum and ocular tissues concertation data. All summary statistics were calculated on unadjusted raw data and then adjusted to three significant figures for reporting purposes. Finally, percent tissue partition (relative ratio of ocular tissue to serum) was estimated using exposure metrics (C_max_ and AUC_o-∞_) in ocular tissues and serum.

## Results

**FIGURE 1:**
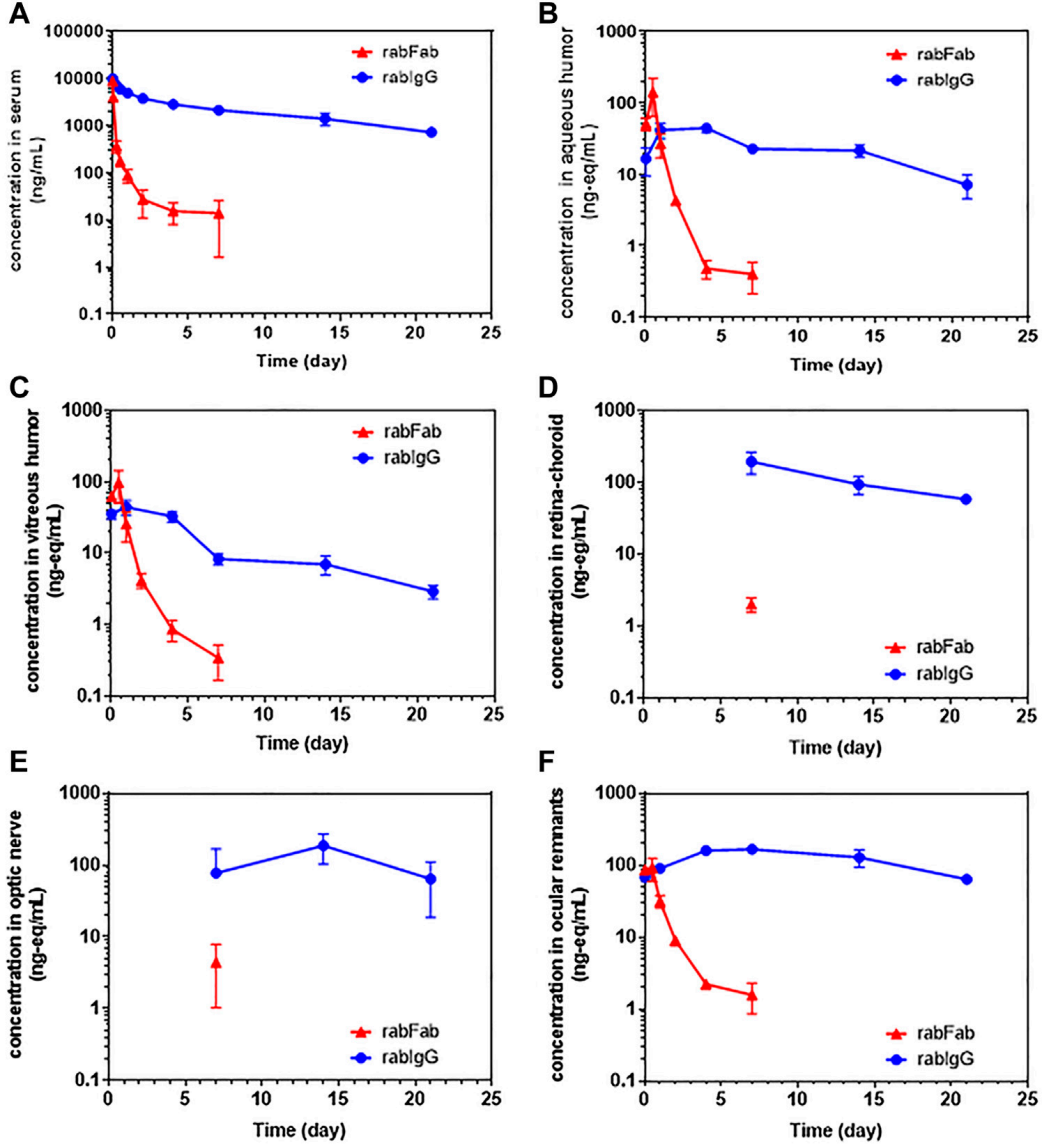
The concentration–time profiles of rabFab and rabIgG in **(A)** serum **(B)** aqueous humor **(C)** vitreous humor **(D)** retina-choroid **(E)** optic nerve, and **(F)** ocular remnants following a single intravenous bolus administration of ^125^I-rabFab or ^125^I-rabIgG (radiolabeled mixed with unlabeled, total 1 mg dose) in male New Zealand White rabbits. Data presented are Mean ± SD. Data in panel **(A)** represent protein bound concentrations in serum reported as nanogram/milliliter (ng/mL) and data in all other tissues (panels **B**-**F)** represent total (protein bound plus catabolized ^125^I) concentrations reported as nanogram-equivalents/milliliter (ng-eq/mL). Concentrations in all matrices are reported in volume units and density of “1” assumed for all weight-based matrices.

Concentration-time profiles of rabFab and rabIgG in serum, aqueous humor, vitreous humor, retina-choroid, optic nerve and ocular remnants are illustrated in Figure 1 and PK parameters are presented in Table 1. In general, both compounds upon single IV bolus dose administration displayed a bi-phasic disposition, with a rapid net distribution phase, followed by slower net elimination phase in serum, as illustrated in Figure 1A. However, the serum profile of rabFab showed a much more rapid decline compared to rabIgG and had overall fast PK (CL = 943 mL/day and t_1/2_ = 1.93 days for rabFab vs. CL = 18.5 mL/day and t_1/2_ = 8.93 days for rabIgG). Similarly, ocular PK (in aqueous and vitreous humor) of rabFab showed a fast absorption phase (T_max_ = 0.5 days) followed by a rapid decline and slow elimination phase, while rabIgG showed a relatively slow absorption (T_max_ = 1–4 days) followed by a slow decline (Figures 1B,C). Similar to observations in serum, rabFab showed fast ocular clearance compared to rabIgG in aqueous and vitreous humor compartments (CL/F = 9060 and 10,900 mL/day and t_1/2_ = 1.57 and 1.43 days vs. CL = 1660 and 2890 mL/day and t_1/2_–8.36 and 9.23 days, respectively). PK assessment in additional compartments of eye such as retina-choroid and optic nerve showed distinct profiles for rabFab and rabIgG (Figures 1D,E) and, if appropriate, PK parameters are provided in Table 1. Quantifiable exposures in retina-choroid and optic nerve were observed only on day 7 (last collection point) for rabFab and from day 7 to day 21 for rabIgG. In general, as seen with other ocular compartments such as aqueous and vitreous humor, rabIgG had higher and prolonged absolute exposure (i.e., observed or measured exposure presented in absolute quantities, e.g., “ng/mL”) compared to rabFab in retina-choroid and optic nerve. Additionally, while rabFab showed comparable PK in ocular remnants (similar to aqueous and vitreous humor), rabIgG showed higher exposure in ocular remnants compared to aqueous and vitreous humor (Figure 1F; Table 1).

**TABLE 1:** Non-compartmental pharmacokinetic parameters of rabFab and rabIgG in serum and ocular compartments following a single intravenous bolus dose of ^125^I-rabFab or ^125^I-rabIgG (radiolabeled mixed with unlabeled, total 1 mg dose) in male New Zealand White rabbits.

Treatment group	Matrix	C_0_ (ng/mL)	C_max_ (ng/mL)	T_max_ (days)	AUC_0-t_ (day*ng/mL)	AUC_0-∞_ (day*ng/mL)	CL or CL/F (mL/day)	V_z_ or V_z_/F (mL)	t_½_ (days)
rabFab	Serum	10,200	8740 ± 277	0.007	1020 ± 66.7	1060	943	2620	1.93
	Aqueous humor^†^	NA	142 ± 38.4	0.50	109 ± 18.8	110	9060	20,500	1.57
	Vitreous humor^†^	NA	97.0 ± 23.4	0.50	90.7 ± 12.1	91.4	10,900	22,500	1.43
	Retina-choroid^†^	NA	2.01 ± 0.234	ND	ND	ND	ND	ND	ND
	Optic nerve^†^	NA	4.45 ± 1.97	ND	ND	ND	ND	ND	ND
	Ocular remnants^†^	NA	92.5 ± 16.3	0.50	111 ± 8.40	116	8620	26,000	2.09
rabIgG	Serum	10,030	9900 ± 214	0.007	44,600 ± 3070	54,000	18.5	239	8.93
	Aqueous humor^†^	NA	44.6 ± 3.15	4.00	516 ± 21.6	602	1660	20,000	8.36
	Vitreous humor^†^	NA	44.5 ± 5.13	1.00	307 ± 15.8	346	2890	38,500	9.23
	Retina-choroid^†^	NA	195 ± 32.7	7.00	2230 ± 248	ND	ND	ND	ND
	Optic nerve^†^	NA	187 ± 42.3	14.0	2080 ± 476	ND	ND	ND	ND
	Ocular remnants^†^	NA	167 ± 5.87	7.00	2650 ± 130	3580^‡^	279	4060	10.1

Data presented are mean ± standard error where applicable; C_0_ – Extrapolated concentration at time “0”; NA - not applicable; ND - PK Parameter could not be calculated or reported due to insufficient data; † - Reported concentration parameters and AUCs represent ng-eq/mL and day*ng-eq/mL, respectively; ‡ - AUC % extrapolated was >20%.

Contrary to absolute exposure differences in serum, relative percent tissue partition (measured based on C_max_ and AUC_0-∞_ in aqueous and vitreous humor to serum) was higher for rabFab compared to rabIgG (Table 2). Specifically, percent tissue partition ranged from 1.11 to 10.4 for rabFab and 0.45 to 1.11 for rabIgG. Similarly, tissue partition in ocular remnants was comparable to aqueous and vitreous humor for rabFab (1.06 and 10.9, respectively), while relatively higher tissue partition was observed in ocular remnants for rabIgG (1.69 and 6.63, respectively). Due to insufficient exposure data to determine AUC_0-∞_ in retina-choroid and optic nerve, percent tissue partition could not be determined in these tissues.

**TABLE 2:** Relative percent ocular tissue:serum partition (C_max_ and AUC_0-∞_ based) of rabFab and rabIgG following a single intravenous bolus dose of^125^I-rabFab or^125^I-rabIgG (radiolabeled mixed with unlabeled, total 1 mg dose) in male New Zealand White rabbits.

Treatment	Matrix	C_max_ based tissue partition (%)	AUC_0-∞_ based tissue partition (%)
rabFab	Aqueous humor	1.62	10.4
	Vitreous humor	1.11	8.62
	Retina-choroid	0.02	ND
	Optic nerve	0.05	ND
	Ocular remnants	1.06	10.9
rabIgG	Aqueous humor	0.45	1.11
	Vitreous humor	0.45	0.64
	Retina-choroid	1.97	ND
	Optic nerve	1.89	ND
	Ocular remnants	1.69	6.63

ND - Parameter could not be calculated due to insufficient data; percent tissue partition = (exposure in tissue (C_max_ or AUC)/exposure in serum (C_max_ or AUC)) × 100.

In addition to serum and ocular compartments, exposure was also assessed in extravascular tissues such as brain, heart, kidney, liver, lungs, intestine and few other additional tissues (Supplementary Figures S1, S2). RabFab had higher exposure in kidney compared to serum (based on C_max_ and AUC) while all other tissues had lower relative exposure. In contrast, rabIgG had the highest exposure in serum among all tissues based on C_max_ and AUC. Furthermore, in contrast to rabFab, rabIgG had a relatively prolonged exposure (higher AUC) in all highly perfused tissues such as liver, lungs, spleen, heart, in addition to kidneys.

Serum TCA precipitation results showed that approximately 96.3–99.6% of radioisotope was protein associated throughout the study period in the case of rabIgG while this value ranged from 51.4 to 97.8% for rabFab (Supplementary Table S1 and Supplementary Figure S3). The higher fraction of non-protein-associated ^125^I for rabFab (up to 50% between 0.25 and 2 days) is presumed to be attributed to the rapid degradation of Fab in kidneys and non-residualizing nature of [^125^I]iodotyrosine and/or associated metabolites. This was further supported by the transient nature of the decrease in protein-associated values and by the higher accumulation of rabFab in kidneys (Supplementary Figures S1, S2). Fab is expected to undergo filtration in kidneys due to small molecular size, followed by eventual reabsorption of non-protein-associated ^125^I (in the form of iodotyrosine and/or other catabolites) post protein degradation of Fab in renal proximal tubules. Due to the higher and more variable fraction of protein unbound ^125^I-containing metabolites in serum for rabFab (Supplementary Table S1), serum concentration data of both rabFab and rabIgG were corrected using TCA-precipitation data prior to PK analysis. However, TCA precipitation analysis in this study was limited to serum data only and neither ocular matrices nor other tissues were subjected for any further correction of concentration data. The percent ocular partition reported for rabFab in this study might be modestly overestimated due to total concentration (i.e., protein bound plus catabolized ^125^I) data used in ocular compartments while more accurate protein bound concentrations used in serum. To understand the likely magnitude of this impact, serum PK of rabFab was estimated using serum total exposure data (Supplementary Table S2) and this serum uncorrected/total PK was used to derive percent relative tissue partition in aqueous and vitreous humor (Supplementary Table S3). Assuming similar proportions and time courses of protein bound and catabolized ^125^I across serum and ocular matrices, it is possible that rabFab may have a slightly lower percentage of relative ocular partition (to what is reported in this work) but this is still higher than that of rabIgG.

## Discussion

This work describes the systemic and ocular pharmacokinetics of rabFab and rabIgG following IV administration in rabbits. We used non-binding, species-matched proteins to test PK and rabbit as our preclinical animal model. Although there are some anatomical differences between rabbit and human eyes (i.e., retinal architecture, physiological volume of vitreous, and retina, vascularization etc.), rabbit is the non-clinical species most commonly used in elucidating ocular pharmacokinetics of drugs, and several reports have showed rabbits to be a reliable preclinical model for assessing ocular PK of drugs and for clinical translation (Del Amo and Urtti, 2015; Ahn et al., 2016).

While the PK of mAbs and Fabs after ITV administration are well established (Crowell et al., 2019; Caruso et al., 2020), no specific reports exists that clearly describe the ocular PK of systemically administered protein therapeutics. Following ITV administration, biologics distribute from vitreous humor into retina (posterior segment of eye) and aqueous humor (anterior segment of eye) and enter the systemic circulation via the choroidal vasculature (though contribution of this route is lower magnitude) and aqueous humor outflow (Lamminsalo et al., 2020); these processes are described to be bidirectional, and are dictated by the convective and diffusive properties of the molecule and physiological environment (Del Amo et al., 2017). The PK behavior of mAbs and antibody fragments in the aqueous humor, retina, and serum post ITV administration typically follow trends similar to that of the vitreous humor, since the drug clearance is rate limited by vitreal elimination (Gaudreault et al., 2005; Bakri et al., 2007b; Gadkar et al., 2015; Del Amo et al., 2017). This is reflected in the PK profiles of ranibizumab and bevacizumab in rabbits where half-lives in vitreous, aqueous humor, and serum compartments were approximately 3 days for ranibizumab and approximately 6 days for bevacizumab (Gaudreault et al., 2007; Sinapis et al., 2011). However, full-length antibodies and fusion proteins with Fc components may have relatively longer systemic half-lives (compared to ocular half-lives) post ITV dosing due to systemic recirculation mediated by neonatal Fc receptor (FcRn). This was evident for bevacizumab where reported aqueous half-life was 9.82 days while serum half-life was 18.7 days in humans post ITV dosing (Krohne et al., 2008; Avery et al., 2017). In general, similar to these reported observations of serum PK post ITV dosing (i.e., comparable half-lives in vitreous, aqueous humor and serum), in this current work, we observed that antibody format, influence of blood ocular barriers (BOB) in ocular distribution of the protein format and serum kinetics govern PK behavior of rabFab and rabIgG in ocular tissues post IV administration.

Exposure-based (AUC_0-∞_) aqueous humor:serum and vitreous humor:serum for rabFab were approximately 10.4 and 8.62% respectively, while for rabIgG were 1.11 and 0.64% respectively (Table 2). The relative partitioning observed for rabIgG of 0.5–1% of systemic concentrations is consistent with reported exposure in fellow eyes following unilateral intravitreal injection of bevacizumab (Bakri et al., 2007b; Miyake et al., 2010). Although, rabFab had higher percent ocular partition (i.e., approximately 2- to 10-fold higher ocular partitioning) compared to rabIgG, absolute exposure in ocular compartments was higher for rabIgG. The relevance and importance of these factors (absolute exposure vs. relative partition to ocular tissues) should be considered on a case-by-case basis in selecting molecular format for clinical candidate selection to minimize ocular exposure and potential toxicities. We emphasize that the higher relative partitioning of rabFab compared with rabIgG is consistent with previous reports regarding the molecular weight dependence of protein transfer from blood to barrier-protected matrices such as BOB where low molecular weight format like Fab can more readily cross these barriers relative to larger formats such as IgG (Grabner et al., 1978; Ragg et al., 2019). On the contrary, higher absolute exposure of rabIgG in ocular compartments is probably driven by three factors; 1) receptor mediated active transport/recycling facilitated by FcRn component of IgG-Fc, 2) extended serum exposure of IgG that drives passive transport to ocular compartments over extended period of time and 3) size-dependent longer vitreous half-life of ocular partitioned IgG compared to smaller size Fab (Crowell et al., 2019) once the IgG is inside the vitreous. However, additional investigations are needed to substantiate these hypotheses. Note that the test compounds in this study (rabFab and rabIGg) differ in their molecular weights by approximately 3-fold (48 vs. 150 kDa). PK data were shown in mass units rather than in molar units, as is typical for the majority of reported literature on these molecule formats. While relative tissue:serum partition is unaffected by choice of units, comparison of absolute exposures between molecules should take into account differences in molar mass. However, the trends reported here for absolute exposures in ocular compartments between rabFab and rabIgG still holds the same, irrespective of units being used (i.e., rabIgG has higher absolute exposure in ocular compartments compared to rabFab).

The magnitude of ocular partitioning of full-length antibody (IgG) observed in our study supports some of the most common reported ocular toxicities for certain anti-cancer therapeutics. In particular, the presence of target protein expression in healthy ocular tissues could drive on-target ocular toxicities as reported with some targeted therapies and ADCs (Renouf et al., 2012; Gan et al., 2015). Ocular toxicities have been reported for ADCs that utilize maytansine derivatives (DM4) or auristatins (MMAF) as cytotoxic payloads (Eaton et al., 2015; Donaghy, 2016). Although it is unclear whether ocular toxicity with ADCs is solely driven by accumulation of unconjugated payload, findings from our work suggest a possible contribution of antibody mediated delivery of cytotoxic payload to compartments within the eye.

In addition to non-specific distribution to ocular tissues, 1) target-related factors such as target expression, abundance and turnover rate in the eye and 2) antibody-related factors such as format, affinity, potency, molecular charge, hydrophobicity and drug-target binding kinetics may influence the magnitude and duration of ocular exposure of IV administered antibodies. In general, molecular attributes such as size, format, charge, hydrophobicity will influence the ocular and systemic PK profiles of ITV dosed protein therapeutics (Crowell et al., 2019). Similarly, these molecular attributes could drive ocular exposure post IV dosing and, if combined with target expression in ocular compartments, may contribute to ocular toxicities reported with protein therapeutics (Advani et al., 2009; Wilson et al., 2016). More comprehensive studies are needed to understand the molecular and target attributes that govern ocular distribution of systemically dosed drugs, with considerations for a given drug format-target pair to adequately capture ocular exposure to understand subsequent ocular safety events.

The findings from this study are limited to Fab and IgG format and may not be directly applicable to other large molecule formats such as (Fab)_2_, scFv, protein-polymer conjugates, as well as other novel formats and treatment modalities such as immunoliposomes, gene and cellular therapy products. Additionally, occurrence of serum anti-drug-antibodies (ADAs) and TMDD are expected to influence systemic exposure and in turn, likely influence ocular exposure. While TMDD is not expected in our study due to non-targeted nature of the test compounds, serum ADAs were not measured as minimal-to-negligible ADA formation was anticipated against species-matched rabFab and rabIgG in the rabbit serum (Shatz et al., 2016).

In contrast to the exposure differences in ocular compartments post local (ITV) administration (vitreous humor > aqueous humor) as shown in Gadkar et al. (Gadkar et al., 2015), we observed comparable exposure in vitreous and aqueous for rabFab across all time points after IV dosing; rabIgG concentrations in vitreous and aqueous were comparable at early time points, after which aqueous concentrations were ∼2 to 3-fold higher than vitreous concentrations. These results for rabIgG distribution are in further contrast to the observations by Bakri et al. (Bakri et al., 2007b) for bevacizumab, which after ITV of fellow eyes, was noted in uninjected eyes to have higher concentrations in aqueous at Cmax, with higher concentrations in vitreous at later time points. This highlights the possibility of lymphatic contribution for entry of drugs into the eye post IV dosing, as well as the potential importance of anterior entry to ocular matrices via the blood-aqueous barrier; the blood-retinal and blood-aqueous barriers, which have distinct blood supplies and cellular architecture, have been noted to operate differently with respect to what molecules may pass through (and under what conditions) (Coca-Prados, 2014).

Quantifiable concentrations of rabFab and rabIgG in retina-choroid and optic nerve in this study were first observed on Day 7, while exposure in vitreous and aqueous humor was quantifiable throughout the study period. Retinal and optic nerve PK assessment is known to be complicated and challenging due to experimental difficulties related to retinal sample collection and sample preparation. Studies that are more definitive are needed to make a clear interpretation of retinal exposure of protein therapeutics and drug partitioning to retina post IV and ITV dosing, as well as to clarify the routes of entry into ocular matrices from circulation. Similarly, exposure in ocular remnants was either similar (rabFab) or higher (rabIgG) than aqueous and vitreous humor but this could be overestimated due to sample preparation procedure and probable contamination of samples with blood since eyes were not perfused prior to collection of ocular tissues. Hence, a clear interpretation of exposure in these ocular compartments was not attempted in this study. Additionally, as described in Results section, ocular exposure data were not corrected for unbound/free radioactivity and this may have a modest impact on the percent ocular partition reported for rabFab. Future studies with antibody bound radioisotope exposure data in serum and ocular compartments or studies that use residualizing radioisotopes such as ^111^In are needed to derive more accurate estimate of percent ocular partition for rabFab. For instance, there is potential for release and partial recirculation of non-protein-associated ^125^I upon renal filtration and degradation of a radioiodinated Fab. In contrast, the ^111^In-containing catabolites of a radiometal-labeled Fab will remain sequestered within lysosomes of renal proximal tubules, rendering them unable to produce artifactually high systemic levels of radioactivity. Additionally, our previous work demonstrated no statistical difference between ^125^I and ^111^In-derived exposure in mice, using AUC_0–7 days_ as a metric, for trastuzumab at doses spanning three orders of magnitude or for a non-binding control antibody (Pastuskovas et al., 2012). This trend of an overall similarity in PK between radioiodinated and radiometallated antibodies has been observed in numerous other studies reported by us and others (Perera et al., 2007). However, we stress that this trend is consistently observed only for full size antibodies with normal interaction with the neonatal Fc receptor. In contrast, Fabs and other small antibody fragments may display disparity between ^125^I and ^111^In signal due to rapid renal filtration, lysosomal degradation, and partial recirculation of non-residualized ^125^I-containing catabolites but not ^111^In-containing catabolites. This was evident in this study with rabFab where measured radioactivity was contributed by both intact (protein bound) and free ^125^I for a short period (between 0.25 and 2 days, see Supplementary Table S1) that may lead to modest overrepresentation of exposure when not corrected for free ^125^I as specified using serum PK data (see Supplementary Table S2).

In conclusion, we characterized ocular and systemic PK of rabFab and rabIgG following IV administration in rabbits and identified that approximately 0.5–10% of systemically administered proteins partition into ocular compartments (aqueous and vitreous humor). Despite its larger size and lower relative percentage of ocular partitioning, full-length antibody (rabIgG) had higher absolute ocular exposure compared to rabFab. Unlike, local (ITV) administration where differences in exposure are expected in ocular compartments (vitreous humor > aqueous humor), comparable exposures were observed in these ocular compartments after systemic administration. This study provides the foundation for understanding ocular PK behavior of antibody-based therapies following IV administration, specifically in predicting format-based relative ocular exposures to predict undesirable pharmacological activities. Findings reported in this work complement our current understanding of ocular PK of systemically administered protein therapeutics and will aid in molecular format selection for clinical candidates to minimize ocular exposure.

## Data Availability

The raw data supporting the conclusions of this article will be made available by the authors, without undue reservation.
