# Supplementary figures and images for: Antibody Format and Serum Disposition Govern Ocular Pharmacokinetics of Intravenously Administered Protein Therapeutics
Source: Front Pharmacol. 2021 May 6;12:601569. doi: 10.3389/fphar.2021.601569 (PMC8138871; doi:10.3389/fphar.2021.601569)

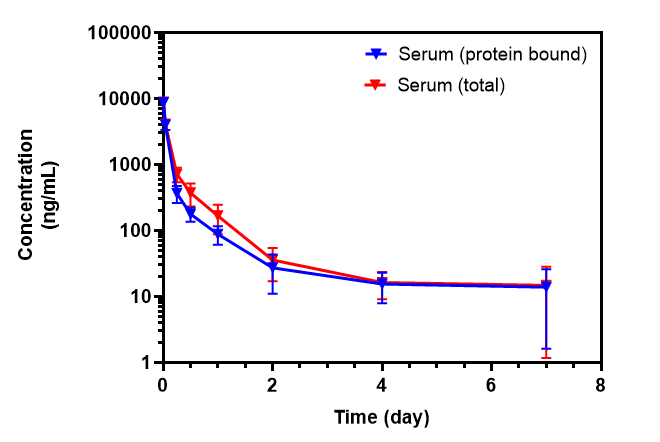

Supplement: Supplementary file 1 [file Image3.TIF]

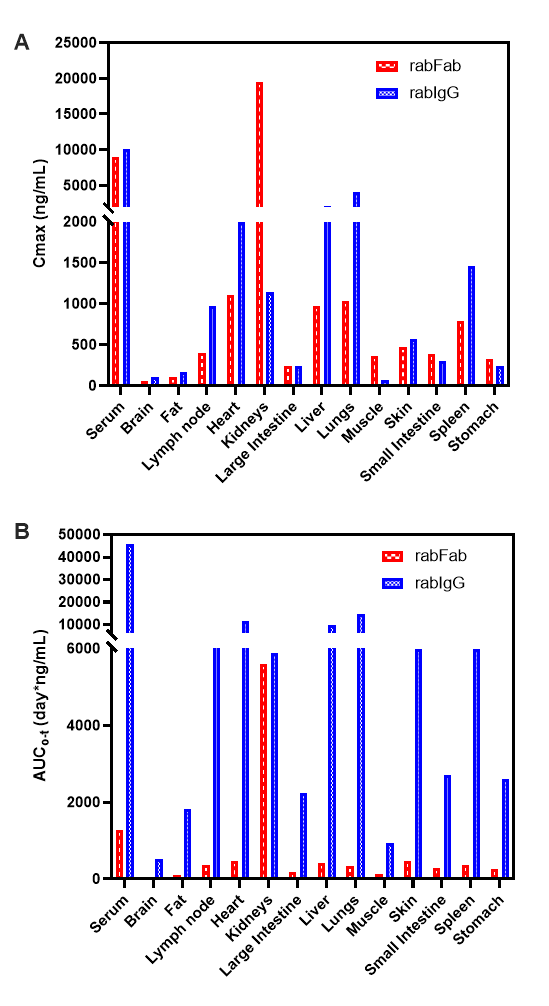

Supplement: Supplementary file 2 [file Image2.TIF]

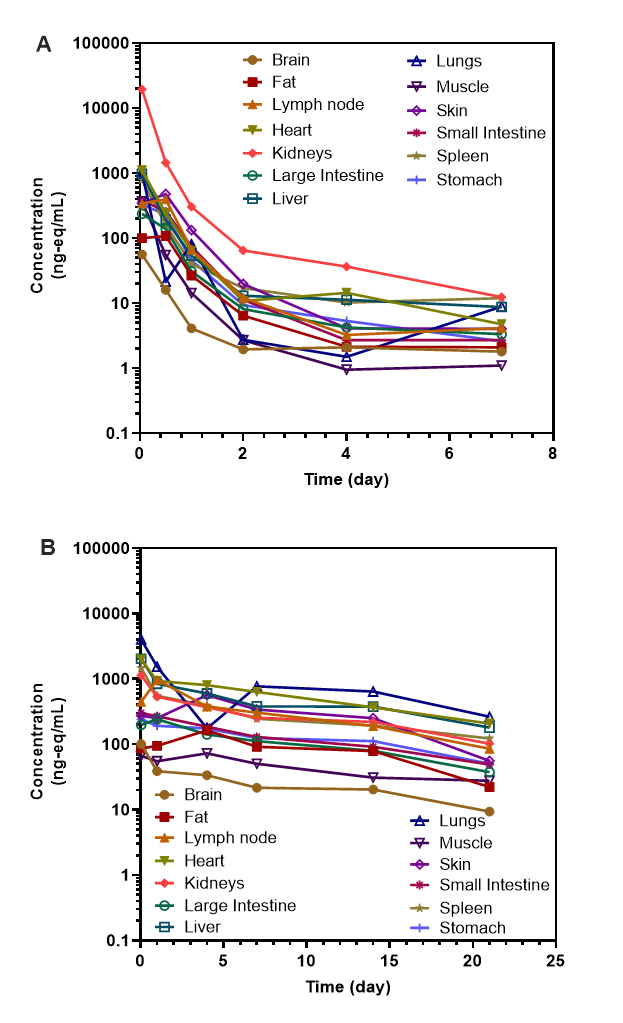

Supplement: Supplementary file 3 [file Image1.TIF]
