# Supplementary material for: Antibody Format and Serum Disposition Govern Ocular Pharmacokinetics of Intravenously Administered Protein Therapeutics
Source: Front Pharmacol. 2021 May 6;12:601569. doi: 10.3389/fphar.2021.601569 (PMC8138871; doi:10.3389/fphar.2021.601569)
Supplement: Supplementary file 4 [file DataSheet1.docx]

**Supplementary Material**

**Supplemental Figure S1.** Pharmacokinetic profiles of rabFab **(A)** and rabIgG **(B)** across various tissues post intravenous bolus administration of ^125^I-rabFab or ^125^I-rabIgG (radiolabeled mixed with unlabeled, total 1 mg dose) in male New Zealand White rabbits. Data in all tissues represent total (protein bound plus catabolized ^125^I) concentrations reported as nanogram-equivalents/milliliter (ng-eq/mL).

**Supplemental Figure S2.** Exposure metrics **(A)** C_max_ and **(B)** AUC_0-t_ across various tissues post intravenous bolus administration of ^125^I-rabFab or ^125^I-rabIgG (radiolabeled mixed with unlabeled, total 1 mg dose) in male New Zealand White rabbits. Data in serum represent protein bound concentrations reported as nanogram/milliliter (ng/mL) and data in all tissues represent total (protein bound plus catabolized ^125^I) concentrations reported as nanogram-equivalents/milliliter (ng-eq/mL). Concentrations in all matrices are reported in volume units and density of ‘1’ assumed for all weight-based matrices. Reported AUCs across tissues (except serum) represent day*ng-eq/mL.

**Supplemental Table S1.** Percent protein bound fractions of rabFab and rabIgG in serum post intravenous bolus administration of ^125^I-rabFab or ^125^I-rabIgG (radiolabeled mixed with unlabeled, total 1 mg dose) in male New Zealand White rabbits (estimated using TCA precipitation of serum aliquots and measurement of radioactivity of supernatant and pellet).

| **Time (days)** | **% protein bound fraction** | |
| --- | --- | --- |
|  | **rabFab** | **rabIgG** |
|  | **Mean ± SD** | **Mean ± SD** |
| 0.007 | 97.8 ± 0.98 | 97.8 ± 1.20 |
| 0.042 | 96.6 ± 0.65 | 98.9 ± 0.53 |
| 0.25 | 51.5 ± 8.55 | 98.3 ± 0.94 |
| 0.5 | 51.4 ± 12.6 | 96.3 ± 3.18 |
| 1 | 56.5 ± 16.9 | 97.2 ± 1.96 |
| 2 | 75.2 ± 16.0 | 98.4 ± 0.50 |
| 4 | 93.9 ± 4.14 | 97.5 ± 0.92 |
| 7 | 96.2 ± 5.41 | 98.2 ± 0.69 |
| 14 | NA | 98.1 ± 1.27 |
| 21 | NA | 99.6 ± 0.35 |

NA – not applicable; % protein bound = (concentration in TCA treated serum pellet/concentration in total serum aliquot) x 100

**Supplemental Figure S3.** The concentration–time proﬁles of rabFab in serum representing protein bound (TCA-corrected) and total (protein bound plus catabolized ^125^I) concentrations, following a single intravenous bolus administration of ^125^I-rabFab (radiolabeled mixed with unlabeled, total 1 mg dose) in male New Zealand White rabbits. Data presented are Mean ± SD.

**Supplemental Table S2.** Non-compartmental pharmacokinetic parameters of rabFab using protein bound (TCA-corrected) and total (protein bound plus catabolized ^125^I) concentration-time data in serum, following a single intravenous bolus dose of ^125^I-rabFab (radiolabeled mixed with unlabeled, total 1 mg dose) in male New Zealand White rabbits.

| Treatment group | Matrix | C_0_  (ng/mL) | C_max_  (ng/mL) | T_max_  (Days) | AUC_0-t_  (day*ng/mL) | AUC_0-∞_  (day*ng/mL) | CL or CL/F  (mL/day) | Vz or V_z_/F  (mL) | t_½_  (days) |
| --- | --- | --- | --- | --- | --- | --- | --- | --- | --- |
| rabFab | serum  (protein bound concentration) | 10200 | 8740 ± 277 | 0.007 | 1020 ± 66.7 | 1060 | 943 | 2620 | 1.93 |
|  | serum  (total concentration) | 10400 | 8940 ± 289 | 0.007 | 1270 ± 92.6 | 1310 | 766 | 1660 | 1.50 |

Data presented are mean ± standard error where applicable; C_0_ - Extrapolated concentration at time ‘0’.

**Supplemental Table S3.** Relative percent ocular tissue:serum partition (C_max_ and AUC_0-∞_ based) of rabFab using protein bound (TCA-corrected) and total (protein bound plus catabolized ^125^I) concentration-time data in serum, following a single intravenous bolus dose of ^125^I-rabFab (radiolabeled mixed with unlabeled, total 1 mg dose) in male New Zealand White rabbits.

| Treatment | Serum data used | Matrix | C_max_ based  tissue partition  (%) | AUC_0-∞_ based  tissue partition  (%) |
| --- | --- | --- | --- | --- |
| rabFab | (protein bound concentration) | aqueous humor | 1.62 | 10.4 |
|  |  | vitreous humor | 1.11 | 8.62 |
|  | (total concentration) | aqueous humor | 1.59 | 8.40 |
|  |  | vitreous humor | 1.08 | 6.98 |

Percent tissue partition = (exposure in tissue (C_max_ or AUC)/exposure in serum (C_max_ or AUC)) x 100
